# The Tosca Registry: An Ongoing, Observational, Multicenter Registry for Chronic Heart Failure

**Published:** 2016-05-16

**Authors:** M Arcopinto, A Salzano, F Ferrara, E Bobbio, AM Marra, R Abete, F Stagnaro, R Polizzi, F Giallauria, M Illario, E Menditto, C Vigorito, E Bossone, A Cittadini

**Affiliations:** 1 IRCSS Policlinico San Donato Milanese, San Donato, Milano; 2 Dipartimento di Scienze Mediche Traslazionali, Università Federico II, Napoli; 3 Ospedale Santa Maria dell’Olmo, Cava de’ Tirreni, Salerno; 4 Pulmonary Hypertension Center, Thoraxclinic, University of Heidelberg, Germany; 5 CIRFF, Center of Pharmacoeconomics, Federico II University, Naples, Italy

**Keywords:** Registry, heart failure, hormones, ageing

## Abstract

The ageing of the population in western countries, the continuous increase of the prevalence of chronic diseases, the frequent coexistence of several morbid conditions (comorbidity) requires health professionals and Institutions to face difficult challenges, including increasing costs, need for more effective and sustainable therapies, and organizational issues.

The European Innovation Partnership on Active and Healthy Ageing aims at enabling European citizens to lead healthy, active and independent lives while ageing.

We herein discuss some key concepts bearing a special significance in the light of the Partnership aims, and present research and educational projects active in our local environment. Among these, the multicentre project TOSCA (Trattamento Ormonale nello Scompenso CArdiaco) that, although primarily focused on the understanding of the interactions between hormones and chronic heart failure (CHF), is also aimed at developing more effective models of clinical care. We provide the scientific background and current stage of the project. In the context of a growing complexity of the patients’ clinical management, the polipharmacy is a new arising challenge for clinicians, bearing direct economic, organizational and clinical implications. A better understanding, characterization and management of this issue represent an additional target of the TOSCA network.

## THE EUROPEAN INNOVATION PARTNERSHIP ON ACTIVE AND HEALTHY AGEING

The European Innovation Partnership on Active and Healthy Ageing will pursue a triple result for Europe: enabling European citizens to lead healthy, active and independent lives while ageing; improving the sustainability and efficiency of social and health care systems; boosting and improving the competitiveness of the markets for innovative products and services responding to the ageing challenge.

This will be realised in the three areas of prevention and health promotion, care and cure, and active and independent living of elderly people. The overarching target of this partnership will be to increase the average healthy lifespan by two years by 2020.

The Partnership aims to achieve this by bringing together all actors in the innovation cycle, from research to adoption, along with those engaged in standardisation and regulation. The Partnership aims at improving the framework conditions for uptake of innovation, improve coordination between funding for social and medical innovation at European, national and regional level in Europe.

This altogether will foster innovation in products, processes and services and, ultimately, will produce benefits for the final users: the older people and care providers [1].

Chronic Heart Failure (CHF) is among the most frequent and complex disease in the elderly, leading to significant functional and social impairment as well as high costs.

In many European countries, > 2% (2.4% in Italy) of the total healthcare budget is related to HF management and up to 70% (80% in Italy) of this cost are related to hospitalizations. In Italy, the pharmaceutical cost represents about 15% of the total health care budget related to HF (150 million Euro/year; 1,3% of the total pharmaceutical cost) [2–3].

Moreover, the recent “device” therapy for HF is further increasing the economic burden for HF management. It has been estimated that an ICD implantation strategy based on current guidelines would increase the rate of implants in Italy by 68% in a 5-year time-frame, impacting heavily on public health system resources. These data suggest the urgent need for effective and better risk stratification to maximize the benefit of ICD therapy and reduce costs in primary prevention [4].

## EPIDEMIOLOGY OF CHRONIC HEART FAILURE

Chronic Heart Failure is a growing public health problem impacting heavily on patient survival, quality of life and healthcare costs. The incidence and prevalence (2–3 of the general population) are clearly increasing in industrialized countries [5–6]. CHF is characterized by (a) shortness of breath at rest or during exertion, and fatigue; (b) signs of fluid retention such as pulmonary congestion or ankle swelling; (c) and an objective evidence of an abnormality of the structure or function of the heart at rest [2].

CHF is a notable exception in the field of cardiovascular disease (CVD). Despite the major improvements obtained in the management of virtually all CVD, CHF still represent a major social and health problem, representing the second most common cause of hospital admission in the world [7] (200.000/year in Italy; 2% of total hospital admissions [8]). CHF is also burdened by unacceptable high hospital readmission rate (approximately 25%) [9], with relevant consequences in terms of health care-cost, patients quality of life and mortality [10–12].

In the ESC-HF Pilot survey, 5118 HF patients (1892 acute HF; 3226 CHF) were enrolled by 136 participating centers in 12 European countries. In the population of acute HF, the survey estimated that the total in-hospital mortality was 3,8% and the length of hospital admission was 8 days [13]. These findings are not congruent to those reported by the registry of the Italian Association of Hospital Cardiologists, ANMCO. In this survey 5643 patients (1868 acute HF; 3775 CHF) were enrolled by 64 participating centers, and estimated a higher in-hospital mortality rate (6,4%) and length of hospital stay (9,78 days) [3,14]. However in both registries (a) in-hospital patients were generally older than ambulatory patients and more often female; 2) comorbidities were more frequent in patients admitted for acute HF, whereas the rate of implanted devices was more common in patients with chronic HF [13].

## HEART FAILURE IN ELDERLY

The prevalence of HF rises with age (from 2–3% to 10–20% at the age of 70–80 years), so that about half of the people with HF are 75 years old [2]. The high prevalence of preexisting structural and functional abnormalities of the heart and that of co-morbidities may explain the worse prognosis of HF in older than in young patients [15]

However, a recent population-based study (2001 residents in Central Italy; age 65–84; prevalence of overt HF 6.7%) estimated a high prevalence of preclinical HF in elderly (59,1%) who do not have risk factors treated at target, suggesting the need of better HF prevention and detection in the older population [16].

Several surveys, including the Euro Heart Failure Survey I (EHFS I) suggest that outcome is particularly poor in elderly patients and that evidence-based therapies are less frequently used and underdosage of recommended medications is constantly found in the elderly [17].

In a national HF audit, age below 75 years was one of the strongest predictors of prescribing evidence-based treatments on discharge, and it is likely that the lower rates of prescribing may contribute to the pooper prognosis in elderly HF subjects.

The overall management of CHF in elderly implies a personalized balance between several factors in order to prescribe the best therapy for the individual patient and to review it at regular timepoints (see paragraphs below).

## COMORBIDITIES AND GUIDELINES ADHERENCE IN ELDERLY

Elderly patients with HF have a high burden of comorbidities and functional and cognitive impairments. Some of these conditions are associated with a greater mortality risk. In a recent study, Authors examined the prevalence of comorbidities and 4 measures of functional and cognitive impairments in 558 participants from the Cardiovascular Health Study who developed incident HF between 1990 and 2002 (mean age of participants 79.2 ± 6.3 years, 52% men). Participants were followed prospectively for more than ten years to determine their mortality risk. Comorbidities were: hypertension, coronary heart disease, peripheral arterial disease, atrial fibrillation, obstructive airway disease, diabetes mellitus, chronic kidney disease, cerebrovascular disease, depression.

Sixty percent of participants had ≥3 comorbidities, and only 2.5% had none. Twenty-two percent and 44% of participants had ≥1 activity of daily living (ADL) and ≥1 instrumental activity of daily living (IADL) impaired, respectively. Seventeen percent of participants had cognitive impairment (modified mini-mental state exam score <80, scores range 0–100). During follow up, 504 participants died, with 1-, 5-, and 10-year mortality rates of 19%, 56%, and 83%, respectively. In a multivariable-adjusted model, the following were significantly associated with greater total mortality risk: diabetes mellitus (hazard ratio [HR]: 1.64), chronic kidney disease (HR: 1.32 for moderate disease; HR: 3.00 for severe), cerebrovascular disease (HR: 1.53), depression (HR: 1.44), functional impairment (HR: 1.30 1 IADL impaired; HR: 1.49 for ≥2 IADL impaired), and cognitive impairment (HR: 1.33). Other comorbidities (hypertension, coronary heart disease, peripheral arterial disease, atrial fibrillation, and obstructive airway disease) and measures of functional impairments (ADLs and 15-ft walk time) were not associated with mortality [18].

Apart from comorbidity, elderly patients present unique clinical attention with regard to frequently unrecognized conditions. For example, elderly hospitalized patients suffer disproportionately from constipation. In a retrospective analysis of elderly patients with CHF exacerbations, failure to use constipation prophylaxis is associated with a significantly longer length of stay [18]. These data suggest that routine use of bowel prophylaxis, a very easy and cheap action, may reduce the duration of hospitalization in elderly CHF patients with preexisting constipation [19]. The fact that elderly patients with heart failure are likely to be vulnerable or frail, demonstrate the importance of a multidisciplinary approach to care.

The therapeutic approaches for HF patients have remained practically unchanged during the last two decades. Following several successful trials in HF, effective treatments in the recommendations of current guidelines have been included. However, several studies showed that guidelines are adopted slowly or inconsistently, often failing to lead to further improvements of patient care quality **(Tab. 1)**.

Underuse and underdosage of medications recommended for heart failure have been specifically demonstrated in the elderly population by the EHFS II [20]. This study showed that a large proportion of patients are not discharged on guidance-indicated treatments.

**Fig. 1.**
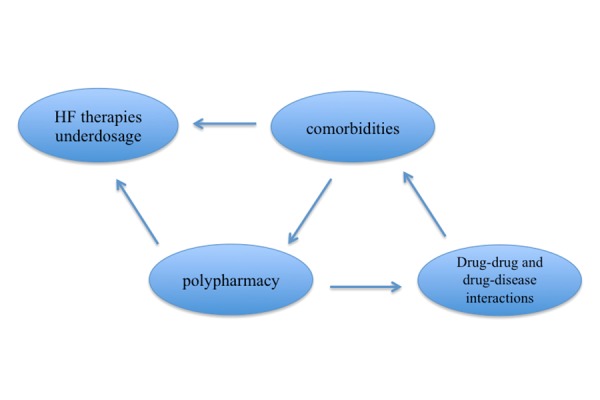
Factors related to HF drug prescription in elderly

The possible reasons for this include a poorer awareness of the use of heart failure treatments in the elderly. Other reasons include the wider prevalence of comorbidities and frailty in the elderly, and increased side effects from medications, which can significantly limit the use of heart failure treatments in the elderly **(Fig. 1)**.

Recent surveys show that the overall guideline adherence is slowly improving over the last decade as results of more effective medical education and physician awareness. With respect to device implantation, the gap between guidelines and practice seems to be greater, probably due to different local medical practice but also to differences in healthcare systems [20].

Thus, one of the most-cost effective ways to reduce the overall burden of HF is the improvement in current guideline adherence.

In this regard, real-world registries based in specific geographical areas are crucial to build a reliable portrait of prescribing performance, device implantation rate, etc.

In the **Table 1**, the most valuable experiences of guideline’s adherence assessment are reported.

Similarly to the mentioned Registy-based studies, the TOSCA project (see below) represent a further opportunity to describe the HF drug prescription pattern in different clinical settings (internal medicine, cardiology, out/inpatients) and to identify major determinants of under- or mis-prescriptions. The yearly comparison of the performances of different Italian HF units may represent a powerful tool for the improvement of the overall medical management in HF. Moreover, it will help to identify the prevalence of hormonal comorbidities and its clinical and prognostic relevance.

## POLIPHARMACY

Polypharmacy is one of the most relevant health-related issues in elderly population. Drug treatment may influence both positively and negatively elderly health status. Polypharmacy increases the risk of inappropriate prescribing, drug–drug and drug–disease interactions, and hence the risk of adverse health events including falls, functional impairment, and hospitalization [20].

Nowadays, different sets of indicators have been developed in order to provide a measure of prescribing performance and, hence, to assess the quality of care in older people. However, this may not be sufficient for frail elderly who have several problems related to the functional status, mobility, cognitive status and living condition. In these patients, the most appropriate approach to re-evaluate the drug-therapy should combine evidence-based data with information gathered from a multidimensional geriatric assessment.

Polypharmacy is associated to a higher hospitalization rate, lower quality of life, increase in health costs, reduction in the patient’s compliance and a lower overall survival.

The network described below is also engaged in the study of this phenomenon. To this aim, we are collecting retrospective and prospective data in order to characterize the pharmacotherapy in patients cared in different centres and settings: inpatients, ambulatory patients; general care vs. Registry-based care.

The systematic review of the drug prescription patterns and comorbidities in our HF patients may reveal common therapeutic practise that are not evidence-based, not associated to any measurable clinical benefit or even harmful for the patients. This might have immediate implications for the real-world practise by:

- reduce inappropriate drug prescription
- arise the awareness for potential drug-drug interactions
- increase the patients engagement in the therapeutic process
- reduce the costs for the patients and the health system

This first large-scale review process may promote the development of special ambulatory structures aimed at critically review the prescribed treatment in patients on polypharmacy.

## THE TOSCA PROJECT

The quest for novel biological systems involved in HF pathogenesis is very active. In this regard, there is increasing evidence that anabolic hormones are down-regulated in CHF patients, thus pointing to a Multiple Hormonal Deficiency Syndrome (MHDS) that represents a reverse model characterized by down- rather than up-regulation of biologically active molecules. The list includes growth hormone (GH) and its tissue effector insulin-like growth factor-1 (IGF-1), androgens, thyroid hormones and insulin axis [30]. Importantly, deficiency of anabolic axes in CHF is consistently associated with impaired functional capacity and poor outcome [31]. In particular, with regard to GH and testosterone, encouraging studies addressing safety and efficacy of hormonal therapies have been carried out [32,33].

Given this background, the TOSCA (Trattamento Ormonale nello Scompenso CArdiaco) Registry is aimed at identify specific patterns of hormonal remodeling in CHF, their correlation with anthropometric and clinical variables, and prognostic implications [34].

The TOSCA Registry is a prospective multicenter observational study designed to evaluate the prevalence of MHDS in CHF patients and its impact on the outcomes of patients affected by CHF. The TOSCA Registry was set up in April 2013 and so far includes 21 centers from all over Italy **(Fig. 3)**. The study is coordinated by the Department of Traslational Medical Sciences - “Federico II” University of Naples (Italy). The study will last for 5 years with an average patients’ follow-up of 3 years. The main outcome measure for this study will be:

- primary end-point: all cause mortality;
- secondary end-point: cardiovascular mortality; hospitalization for decompensated HF.

Patients will be enrolled with the following inclusion criteria:

1. patients of both sexes affected by CHF, diagnosed following current ESC guidelines;
2. left ventricular ejection fraction less than 40%
3. written informed consent.

Exclusion criteria will be:

1. end-stage renal disease requiring dialysis treatment
2. liver cirrhosis class C (Child Pugh)
3. active malignancy
4. active autoimmune disease
5. acute coronary syndrome in the previous 6 months.

All patients will be on top of optimal therapy according to current guidelines.

**Fig. 2.**
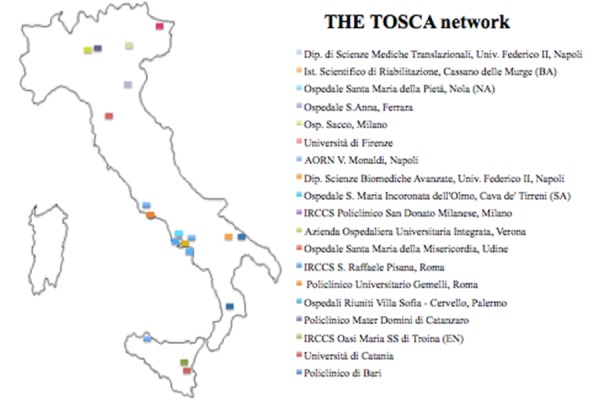
The TOSCA network

**Fig. 3.**
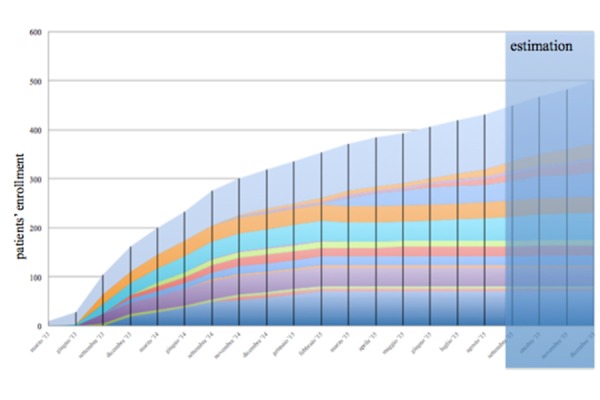
Enrollment rate in the TOSCA Registry

This ambitious program is made possible with the effort of a well-matched national network involving more than 20 centres **(Fig. 2)**.

Patients’ recruitment has started at the coordinating center on April 2013 and has been gradually extended to the other participating centres. The figure below depicts the trend of the CHF patients’ enrolment to date, and the estimated total enrolment at the end of 2015.

Patients enrolled in the TOSCA Registry will undergo a clinical, instrumental, as well as blood work including five sample of serum stored at −80°C at baseline, and subsequently every year. One sample out of five will be employed for centralized measurement of hormones targeted by the study (testosterone, IGF-1, DHEA-S, insulin) while the others will be stored for subsequent analysis to explore new potential features of the CHF pathophysiology.

We think the TOSCA Project will significantly contribute to the aims of the European Innovation Partnership on Active and Healthy Ageing by providing new tools prognostic stratification in adult and elderly HF patients. Moreover, the TOSCA project can be considered a multifunctional platform with education opportunities for HF specialists, cardiologists, internists. During its early years, several TOSCA-sponsored events have been held in different centres belonging to the TOSCA network. These meeting will serve many purposes:

1. To periodically update on the ongoing scientific projects
2. To encourage the young professionals engagement in scientific activities
3. To review and discuss pertinent international guidelines
4. To share HF care models from different setting and environment (hospital/university/outpatients)

To further strengthen the educational mission of the TOSCA project, a stable partnership with the “The Mediterranean School of Cardiovascular Sciences” has been established. The School, founded in Amalfi in October 2011, represents a crossroads of free knowledge and cultures in the heart of the Mediterranean and is at its second edition (October 2014). The dedicated website is http://www.med-school.info/2015/it/ [35]. In this portal, there is a special section for the TOSCA projects, including upcoming events, monthly newsletter, slide sets, news, and project progress.

## Figures and Tables

**Tab. 1.** Registries evaluating therapy adherence in CHF.

**Registry name [ref]**	**Country**	**n.pts**	**Indicators/main findings**
**ALERT-HF [21]**	Italy	660	Medical and device prescription. Poor adherence to therapeutic guidelines with regard to aldosterone antagonists, anticoagulant therapy in the presence of atrial fibrillation and to the use of implantable cardiac devices.
**FUTURE survey [22]**	France	1137	Medical prescription. There were no major differences in treatments and dosages between the groups with low and preserved LVEF.
**ESC-HF Long-Term Registry [17]**	Europe	12 440	Medical and device prescription. About 30% of patients received the target dosage of these drugs. A documented reason for not achieving the target dosage was reported in almost two-thirds of them.
**Heart Failure Adherence Retention Trial (HART) [23]**	USA	692	Medical prescription. Adherence to evidence-based therapy is less than optimal in HF patients based on a combination of both physician and patient nonadherence.
**GWTG-HF registry [24]**	USA		Medical prescription. Patients with increased severity of renal dysfunction were less likely to receive important guideline-recommended therapies.
**MAHLER-Studie [25]**	Europe	1410	Medical prescription. Adherence of physicians to treatment guidelines is a strong predictor of fewer CV hospitalizations.
**Heart Failure Survey in Israel [26]**	Israel	4102	Medical prescription. The use of angiotensin-converting enzyme/angiotensin receptor blockers and beta blockers both declined from NYHA class I to IV.
**IMPROVE HF [27]**	USA	15381	Medical and device prescription. The Registry to Improve the Use of Evidence-Based Heart Failure Therapies in the Outpatient Setting was associated with substantial improvements in the use of guideline-recommended therapies in outpatients with HF.
**IMPACT RECO survey [28]**	France	1919	Medical and device prescription. Prescription rates of CHF drugs were higher than previously reported. Dosages were lower than those recommended in guidelines. Age remained an independent predictor of under-prescription of CHF drugs.
**ODIN [29]**	France	3237	Medical prescription. Age and type of CHF (reduced versus preserved) appeared to be important factors in lack of adherence to guidelines.
